# Plasma miR-34a-5p outperforms miR-126-3p in predicting cognitive decline in cerebral small vessel disease patients with impaired glucose regulation

**DOI:** 10.3389/fnagi.2026.1807882

**Published:** 2026-06-11

**Authors:** Yanan Wang, Baoying Sun, Hui Li, Yuanyuan Ma, Yan Wang, Yijun Song

**Affiliations:** 1 Tianjin Medical University General Hospital, Tianjin, China; 2 Department of Neurology, Affiliated Hospital of North China University of Science and Technology, Tangshan, Hebei, China; 3 Fengnan District Hospital, Tangshan, Hebei, China; 4 State Key Laboratory of Experimental Hematology, National Clinical Research Center for Blood Diseases, Haihe Laboratory of Cell Ecosystem, Institute of Hematology and Blood Diseases Hospital, Chinese Academy of Medical Sciences and Peking Union Medical College, Tianjin, China; 5 Tianjin Institutes of Health Science, Tianjin, China

**Keywords:** cerebral small vessel disease (CSVD), cognitive decline, impaired glucose regulation (IGR), miR-126-3p, miR-34a-5p

## Abstract

**Introduction:**

MicroRNAs (miRNAs) are promising biomarkers for the diagnosis and prognosis of neurodegenerative diseases. Over the past few years, miR-34a-5p and miR-126-3p have become some of the most characterized miRNA, the former being associated with cellular senescence and apoptosis and the latter with the maintenance of vascular endothelial function. The present study aimed to evaluate diagnostic performance of miR-34a-5p and miR-126-3p for cognitive dysfunction in CSVD patients. In addition, we investigated whether these miR-34a-5p and miR-126-3p have mediating effect of impaired glucose regulation (IGR) status on cognitive dysfunction. The final objective was to the identification of better diagnostic biomarkers and further investigation on their value of practical application in Chinese population.

**Methods:**

The study included 300 patients with cerebral small vessel disease (CSVD), who were further stratified by oral glucose tolerance test into those with impaired glucose regulation (IGR, *n* = 151) and those with normal glucose metabolism (NGM, *n* = 149), with the NGM group serving as the internal control. Concurrently, all patients were stratified according to Montreal Cognitive Assessment scores into cognitive function subgroups (normal/mild/moderate/severe impairment). Plasma levels of miR-34a-5p and miR-126-3p were measured in all participants using reverse transcription-polymerase chain reaction (RT-PCR). Mediation analysis was employed to assess the mediating role of the relevant miRNAs in the pathway from IGR to cognitive dysfunction in CSVD patients. The diagnostic performance of these miRNAs for cognitive function and long-term prognosis in CSVD patients with IGR was compared in terms of specificity, sensitivity, and accuracy. Moreover, the miR-34a-5p target genes were screened, and functional and pathway enrichment analyses were performed.

**Results:**

The results show that, in the analysis based on cognitive stratification, plasma miR-126-3p expression levels were negatively correlated with the severity of cognitive impairment in CSVD patients, whereas miR-34a-5p levels showed a positive correlation. miR-126-3p exhibited a stepwise decrease with increasing cognitive severity, while miR-34a-5p significantly distinguished between normal and impaired cognitive status. Mediation analysis revealed that only miR-34a-5p demonstrated a significant mediating effect in the pathway from IGR to cognitive dysfunction (indirect effect = 0.4519, 95% BootCI: 0.0277–0.8879), and it exhibited superior diagnostic performance for cognitive impairment within the CSVD cohort. Following evaluation using the Youden index, miR-34a-5p at the 2-year follow-up demonstrated a balanced diagnostic performance with an area under the curve (AUC) of 0.803, a sensitivity of 65.3%, and a specificity of 84.0% at the optimal cut-off of 2.49, indicating its potential utility for risk stratification. Compared with its performance at baseline (AUC: 0.679, sensitivity: 58.3%, specificity: 75.0%), the diagnostic performance of miR-34a-5p at the 2-year follow-up showed improvements across all metrics, particularly in specificity. In contrast, miR-126-3p (an inverse indicator) showed a moderate sensitivity (64.6–70.5%) and specificity (60.7–63.2%), which may limit its standalone diagnostic application. Enrichment analysis indicated that miR-34a-5p could be involved in the pathological aspects of cognitive impairment by regulating pathways related to apoptosis, oxidative stress, and synaptic function.

**Discussion:**

The IGR stage may represent a potential early pathological window of cognitive impairment in CSVD patients. Plasma miR-34a-5p is more valuable as diagnostic and long-term prognostic marker in IGR patients than miR-126-3p and may mediate association between IGR and cognitive decline via apoptosis, oxidative stress and synaptic pathway.

## Introduction

According to researchers’ estimates (Duering et al., 2023), in the year 2050, there will be 132 million dementia cases around the world. Vascular factors account for a large part and cerebral small vessel disease CSVD is a prime example of this. The study revealed that CSVD is responsible for 45% of dementia cases globally. Furthermore, it is the second most common type of dementia after Alzheimer’s disease (Wardlaw et al., 2019; Pinheiro et al., 2025). The term cerebral small vessel disease (CSVD) refers to a pattern of lesions and damage in the brain caused by the partial or complete blockage of small cerebral arteries (typically with a diameter of less than 40 mm) (Long et al., 2025). These vascular changes will slowly damage the anatomy of the brain. According to Palta et al. (2021), such damage can be visualized as white matter hyperintensities (WMH), covert brain infarction (CBI), cerebral microbleeds (CMB), cortical superficial siderosis (cSS), and MRI-visible perivascular spaces (PVS) on MRI (in a new or different field). An effective indicator that can predict cognition dysfunction due to CSVD is yet to be determined.

Type 2 diabetes is a common condition that can have serious complications if left untreated. As many as 40 % of people with T2DM have brain scans that show signs of CSVD – such as white matter hyperintensities or microbleeds – and the more pronounced these are, the poorer the cognitive performance (Wardlaw et al., 2013). Oxidative stress resulting from hyperglycemia may represent an etiological pathway linking diabetes to CSVD (Yamagishi et al., 2008). Nevertheless, it remains unknown whether impaired glucose regulation (IGR) can serve as a therapeutic time window for cognitive dysfunction in CSVD patients.

A clinical manifestation of CSVD often occurs insidiously with complex and non-specific presentations. In these patients’ cognitive function’s assessment, clinicians primarily focus on assessments of cerebral small vessel structure/function and cognitive scales. According to some studies, if practitioners rely on symptoms and scale scores, around a quarter of vascular dementia (VaD) cases might be misdiagnosed (Rabinovici et al., 2019). A recent study has shown that early intervention in high-risk groups can impede the risk of CSVD cognitive dysfunction developing in the patient (Wardlaw et al., 2019). We need sensitive and reliable tests to identify patients who are most likely to benefit from early intervention.

MiRNAs are small non-coding regulatory RNAs, 2–25 nucleotides long (Long et al., 2025). The uncontrolled regulation of miRNAs is one of the contributors to cognitive impairment. According to Woldemichael and Mansuy (2016), these small non-coding RNAs carryout their regulation through either or both transcriptional or translational pathways. Micro RNA (miRNA) has a high abundance in neurons and glial cells especially in dendrites and synapses. Moreover, their level is tightly regulated with an activity dependent manner. Thus, dysregulation of miRNAs may interfere with neuronal-related processes, they being learning as well as memory formation (Barter and Foster, 2018). MiR-34a-5p was observed to be significantly upregulated in cognitive impairment of diabetics (Liu et al., 2024). Synaptic dysfunction is one of the most important pathologies of cognitive impairment. One study concluded that, removing miR-34a from the genome of APP/PS1 mice caused their cognitive function to improve. The levels of receptor expression were significantly increased in the APP/PS1 mice without the miR-34a-5p (Bahlakeh et al., 2021). MiR-34a-5p regulates the expression of NMDA receptor genes such as Grin1, Grin2a, and Grin2b (Xu et al., 2018) and alters synaptic strength, with synaptic functional integrity being crucial for the maintenance of normal cognitive function. After vascular cognitive impairment (VCI), serum levels of MiR-126 decrease, which regulates vascular function and promotes vascular integrity and angiogenesis (Grossi et al., 2021; Chum et al., 2022). The aforementioned study established a VCI mouse model, where miR-126 downregulation led to inflammation, decrease in myelin and axonal density, impairment of lymphatic and aquaporin function, and a consequent reduction in cerebral blood flow and cognitive function (Yu et al., 2019).

At present, most of the cognitive and miRNA research are focused on Alzheimer’s disease, while the vascular cognitive impairment (VCI) is responsible for 45% of dementia. However, comprehensive *in vitro* and *in vivo* studies remain insufficient. The VCI related to CSVD (Cerebral Small Vessel Disease) is a problem which onset is insidious onset with many forms. It is common to use Type 2 diabetes mellitus (T2DM) as the intervention window in past studies, but the optimal intervention time may be missed. Furthermore, the existing predictive indicators are not enough, especially for the long-term prognosis of cognitive function of CSVD patients. In fact, there is an urgent need for convenient, sensitive, and reliable indicators which are essential to guide clinical practice and also prevent excessive testing and evaluation that can cause patient suffering. This study aims to provide a suitable intervention time window and the ideal choice of biomarkers for cognitive function intervention in CSVD which can avoid the economic burden of the patients.

## Methods

### Study design and participants

This investigation was regarded a prospective observational cohort study in a tertiary hospital from 2021 to 2023. A total of 300 patients with a diagnosis of cerebral small vessel disease (CSVD) were consecutively enrolled. Using a Philips Ingenia 3.0 T system, cranial magnetic resonance scanning was performed on all subjects. Routine clinical physical examination, neuropsychological assessment, and plasma miRNA detection were performed. The Ethics Committee of our hospital approved the study protocol (Approval No. 201904230006). Prior to enrollment, all participants provided voluntary written informed consent.

First-layer stratification based on cognitive function: The Montreal Cognitive Assessment (MoCA) score of 26 was adopted as the cutoff value for normal cognitive function, the scores were adjusted for education level according to established norms prior to group categorization. All patients were divided into the normal cognitive group (*n* = 163, MoCA ≥ 26) and cognitive impairment group n=137,MoCA<26 . Patients with cognitive impairment were further subdivided into mild cognitive impairment subgroup (*n* = 67, MoCA 18–25), moderate cognitive impairment subgroup (*n* = 53, MoCA 10–17), and severe cognitive impairment subgroup n=12,MoCA<10 (Hachinski et al., 2006). Second-layer stratification based on glucose metabolism: In accordance with the 2006 World Health Organization (WHO) oral glucose tolerance test diagnostic criteria, all participants were classified into the normal glucose metabolism (NGM) group n=149 and impaired glucose regulation (IGR) group n=151 . All participants received standardized 2 years of clinical follow-up, andchange in MoCA score (ΔMoCA) defined the main longitudinal outcome indicator.

### CSVD imaging assessment

All cranial imaging analyses were according to the 2023 Standards for Reporting Vascular Changes on Neuroimaging 2(STRIVE-2) (Duering et al., 2023). CSVD enrollment eligibility criteria included presenting at least one positive imaging manifestation (including at least WMH, lacunar infarction, cerebral microbleeds, or EPVS) for patients. The severity of WMH was evaluated by the Fazekas scale. To evaluate the deep white matter, the following score were used: The scorings were 0 = no hyperintense areas, 1 = punctate hyperintense areas, 2 = partially confluent hyperintense areas, 3 = large confluent hyperintense areas. For periventricular white matter, scores were defined as: 0 = no hyperintensity; 1 = cap-like or pencil-thin lining; 2 = smooth halo extending into periventricular region; 3 = irregular hyperintensities extending into deep white matter. All subjects underwent diffusion-weighted imaging (DWI) and magnetic resonance angiography (MRA) to exclude acute cerebral infarction (ACI) within 2 weeks and intracranial macrovascular stenosis (stenosis rate ≥ 50%). The severity of the overall CSVD pathology was quantified using the Staals total burden score (Staals et al., 2014). It consists of total score which ranges from 0 to 4. Each imaging feature which was positive was scored 1 point including lacunar infarction, and moderate to severe WMH (deep Fazekas ≥2 or periventricular Fazekas = 3). Deep or infratentorial microbleeds and moderate to seere basal ganglia EPVS (quantity >10). Higher scores indicated a more severe overall pathological burden of CSVD.

### Inclusion and exclusion criteria

Inclusion criteria: patients aged 60–80 years; definite CSVD imaging abnormalities confirmed by 3.0 T cranial MRI; complete baseline clinical, laboratory and neuropsychological data.

Exclusion criteria: newly diagnosed acute cerebral infarction within 2 weeks or intracranial macrovascular stenosis ≥ 50% confirmed by DWI and MRA; typical clinical and imaging manifestations suggestive of Alzheimer’s disease or other neurodegenerative diseases with medial temporal lobe atrophy (MTA ≥ grade 2) based on the NIA-AA diagnostic criteria (Garnier-Crussard et al., 2020), severe mental disorders, thyroid dysfunction, severe hepatic and renal insufficiency, or other systemic diseases that independently cause cognitive decline; previously diagnosed type 2 diabetes; poor image quality that failed standardized CSVD imaging evaluation.

### Clinical covariates and cognitive function assessment

Baseline demographic and clinical data were collected, including age, gender, years of education, total CSVD burden score, history of hypertension, stroke history, smoking status, drinking status, aspirin and statin medication history, and body mass index (BMI). The 15-item Geriatric Depression Scale (GDS-15) (Zhang et al., 2022) was used to assess baseline depressive symptoms. Cognitive function was evaluated using the MoCA scale at baseline (T0) and 2-year follow-up endpoint (T2). The cognitive change value was calculated as ΔMoCA = MoCA score at T2 − MoCA score at T0.

#### Plasma miRNA processing and quantitative real-time PCR detection

Venous blood from fasting patients was stored in sodium citrate vial. After incubation at room temperature (approximately 25 °C) for 2 h, samples were centrifuged at 1,200 × g and 4 °C for 15 min to separate plasma. The plasma was instantly divided into sterile cryotubes and kept at −80 °C until it was analyzed. Total RNA was extracted from 50 μL of thawed plasma using Trizol LS reagent (Invitrogen). Before RNA extraction, a synthetic *Caenorhabditis elegans* miRNA (cel-miR-39-3p, 1 × 10^8^ copies) was spiked into each sample as an exogenous control to normalize for variations in RNA extraction and reverse transcription efficiency. Reverse transcription and quantitative real-time PCR (qRT-PCR) were performed using the Bulge-Loop™ miRNA qRT-PCR kit. The mature miRNA sequences (from miRBase) were as follows: hsa-miR-126-3p: 5′-ucguaccgugaguaauaaugc-3′; hsa-miR-34a-5p: 5′-uggcagugucuuagcugguugu-3′. Corresponding DNA primers were synthesized based on these sequences. The exogenous control cel-miR-39-3p exhibited consistent amplification across all samples (mean Ct ± SD: 26.98 ± 1.14; coefficient of variation: 4.2%), therefore, technical reproducibility was ensured. The expression level of each target miRNA was evaluated through the 2^-ΔCt^ method, wherein the ΔCt is depicted as Ct (target miRNA) – Ct (cel-miR-39-3p). No further normalization was done to endogenous controls. Samples were analyzed in triplicate, and the average Ct value was used for computations. The MIQE guidelines (Bustin et al., 2009) was followed to design and report all procedures.

#### Follow-up protocol

All participants were given standardized clinical follow-up of 2 years (±1 month). Cognitive assessments were made at baseline (T0) and the endpoint of 2 years follow-up (T2). The ΔMoCA was taken as the primary longitudinal outcome. Detection of plasma miRNA was only performed at baseline and was not repeated. Of the first 300 enrolled CSVD cases, 7 were lost to follow-up. The statistical analysis ultimately involved 293 participants with complete paired baseline and follow-up MoCA data.

### Target gene screening and functional enrichment analysis

Utilizing the miRWalk database, the downstream target genes of hsa-miR-34a-5p were predicted, obtaining results in the human species only, 3′UTR binding site, and prediction score above 0.95. Wet laboratory verification objectives genes screened further from miRTarBase were flagged high confidence target genes. The imported validated target gene symbols were subjected to Metascape human-specific enrichment analysis. Gene Ontology analysis of molecular function and Kyoto Encyclopedia of Genes and Genomes pathway enrichment analysis with the threshold of *p* < 0.01 was performed for statistical significance. The Metascape platform automatically generated GO bar charts, KEGG bar charts, and functional pathway network diagrams, whereby the enrichment significance was visualized by −log10(P).

### Statistical analysis

SPSS 27.0 software and PROCESS Macro v4.2 were used for statistical analysis. Baseline characteristics between groups were compared according to data distribution. Non-normally distributed continuous data were presented as median with interquartile range, and group differences were analyzed using the Kruskal-Wallis test. Multivariate logistic regression analysis was applied to screen independent influencing factors for CSVD-related cognitive impairment. Mediation analysis was performed using Hayes’ PROCESS Model 4 with 5,000 Bootstrap resamples to calculate the 95% confidence interval of indirect effects. Receiver operating characteristic (ROC) curves and the corresponding area under the curve (AUC) were plotted to evaluate the diagnostic sensitivity and specificity of target miRNAs for CSVD-related cognitive impairment.

## Results

### Differential analysis of indicators across groups

Compared to the cognitively unimpaired (CUC) group, the cognitive impairment group showed significantly higher levels of miR-34a-5p P=0.003 , total cholesterol (TC) (*p* < 0.001), and was more likely to be female P=0.029 , while miR-126-3p P=0.001 , CSVD total burden score (*p* < 0.001), and aspirin use P=0.036 were significantly associated with lower odds of cognitive impairment (all *p* < 0.05, Table 1).

**Table 1:** Multivariate logistic regression analysis of factors related to cognitive impairment in CSVD patients with IGR.

Variables	*B*	*SE*	Wald	*p*	Exp (B)	95% CI for Exp (B)
miR-126-3p	−0.239	0.073	10.826	0.001	0.787	0.683—0.908
miR-34a-5p	0.167	0.056	8.808	0.003	1.182	1.058—1.319
Education (EDU)	−0.011	0.037	0.096	0.757	0.989	0.920—1.062
CSVD total burden score (burden)	0.569	0.138	17.097	<0.001	1.767	1.349—2.315
Total cholesterol (TC)	−0.568	0.147	14.852	<0.001	0.567	0.424—0.756
Low-density lipoprotein cholesterol (LDLC)	−0.149	0.206	0.52	0.471	0.862	0.576—1.291
Age	0.029	0.024	1.392	0.238	1.029	0.981—1.080
Aspartate aminotransferase (AST)	−0.012	0.024	0.263	0.608	0.988	0.942—1.035
Alanine aminotransferase (ALT)	−0.006	0.014	0.203	0.652	0.994	0.968—1.021
Gender (1 = Male, 2 = Female)	−0.649	0.298	4.739	0.029	0.523	0.291—0.937
Hypertension (HTN)	−0.034	0.297	0.013	0.909	0.967	0.540—1.732
Coronary artery disease (CAD)	−0.477	0.311	2.347	0.125	0.621	0.337—1.143
Smoking (1 = Yes, 0 = No)	0.152	0.313	0.237	0.626	1.165	0.631—2.150
Alcohol (1 = Yes, 0 = No)	−0.249	0.324	0.587	0.444	0.78	0.413—1.473
Triglycerides (TG)	−0.339	0.192	3.112	0.078	0.712	0.489—1.038
High-density lipoprotein cholesterol (HDL)	0.501	0.346	2.101	0.147	1.65	0.838—3.249
Stroke	−0.169	0.338	0.251	0.616	0.844	0.436—1.636
Aspirin (1 = Yes, 0 = No)	−0.712	0.34	4.386	0.036	0.491	0.252—0.955
Statin (1 = Yes, 0 = No)	0.262	0.36	0.529	0.467	1.299	0.642—2.629
Body mass index (BMI)	0.001	0.038	0	0.988	1.001	0.929—1.078
Impaired glucose regulation (IGR)	−1.697	0.338	25.185	<0.001	0.183	0.094—0.355

### Association analysis of miR-34a-5p and miR-126-3p expression levels with baseline and follow-up cognitive severity grades

**Figure 1:**
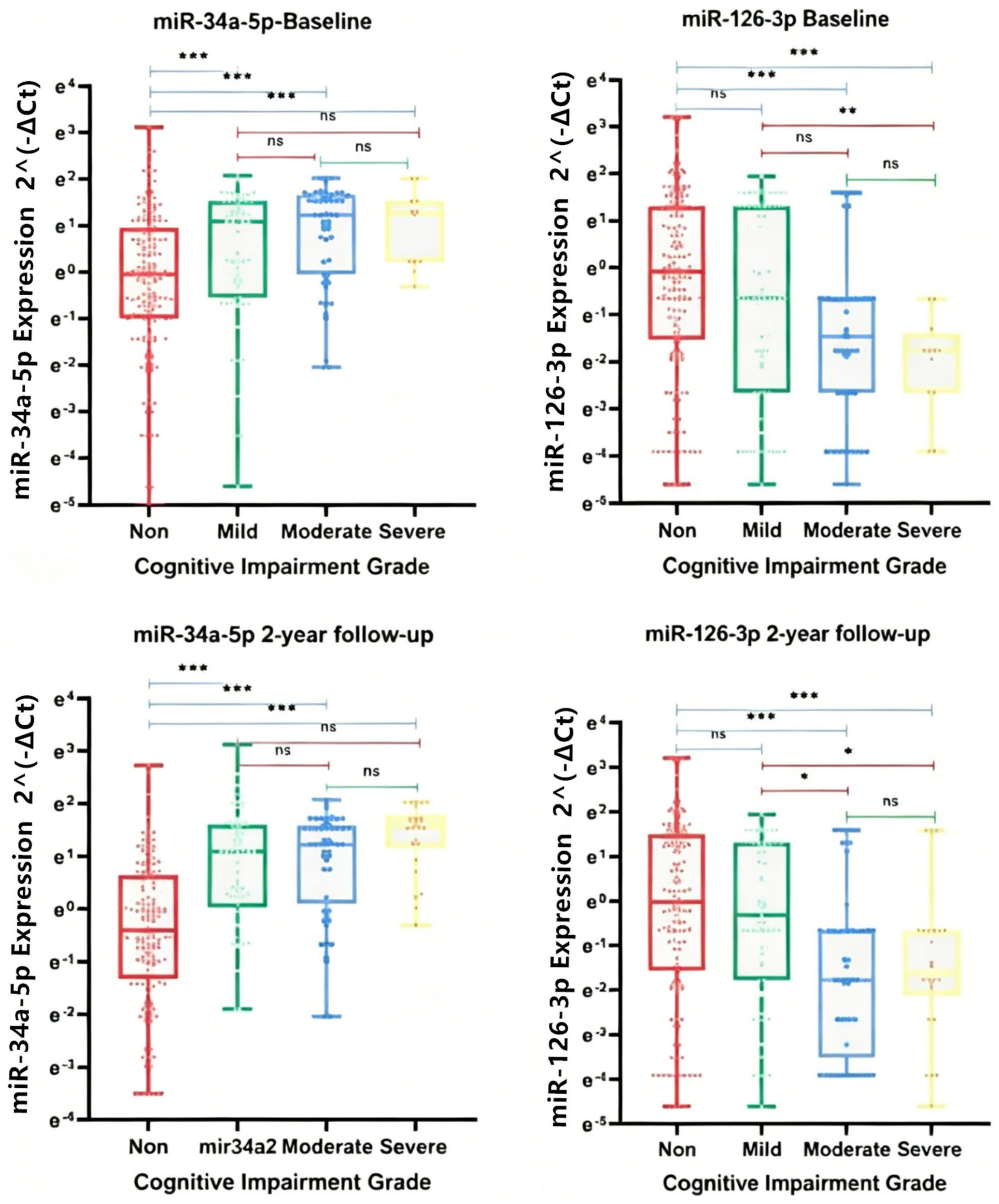
Correlation of miR-34a-5p and miR-126-3p expression with baseline and follow-up cognitive severity grades.

The results (Figure 1) showed that miR-34a-5p expression was significantly correlated with patients’ baseline (*H* = 30.404, *p* < 0.001) and 2-year follow-up (*H* = 31.465, *p* < 0.01) cognitive function, but was not correlated with the severity of cognitive decline (among mild, moderate, severe groups). miR-126-3p expression was significantly correlated with patients’ baseline (*H* = 21.793, *p* < 0.001) and 2-year follow-up (*H* = 19.783, *p* < 0.01) cognitive function, and showed a stepwise decreasing trend with increasing severity of cognitive decline (mild → moderate → severe), indicating a correlation with the severity of cognitive decline.

All data were analyzed using the Kruskal-Wallis test followed by Dunn’s multiple comparisons test. All comparisons were adjusted with the Bonferroni correction. Significance is denoted as follows: ***p < 0.001, **p < 0.01, *p < 0.05, ns (not statistically significant).

### Mediation effect analysis of IGR affecting cognitive impairment in CSVD

**Figure 2:**
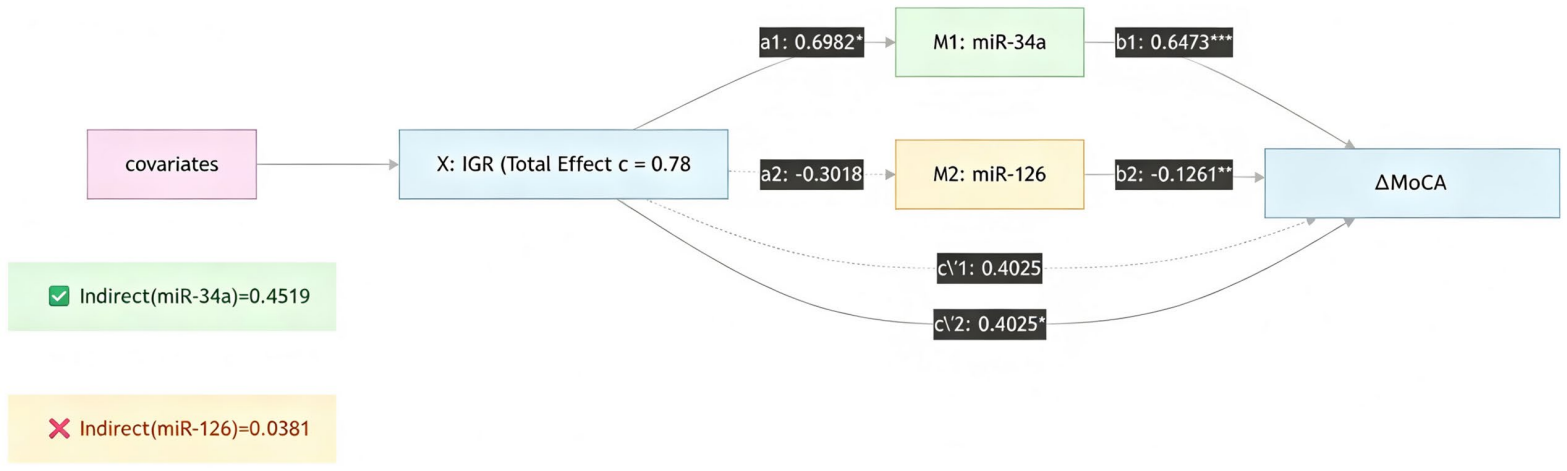
Path diagram of the mediation analysis. This path model showed the mediating effects of miR-34a-5p and miR-126-3p on the association between impaired glucose regulation and 2-year changes in MoCA score (ΔMoCA). All analyses were adjusted for relevant clinical covariates.

In a mediation model with the change in MoCA score (ΔMoCA) as the dependent variable (Y), impaired glucose regulation (IGR) as the independent variable (X), and miR-34a and miR-126-3p as separate mediator variables (M), we obtained the following key findings after controlling for covariates such as age, gender, education, cerebrovascular burden, hypertension, stroke history, aspirin, statin, smoking, alcohol consumption, baseline depressive symptoms and BMI. MiR-34a showed a significant mediating effect between IGR and cognitive impairment (indirect effect = 0.4519, 95% BootCI: 0.0277–0.8879, excluding 0), while miR-126-3p showed no significant mediating effect (indirect effect = 0.0381, 95% BootCI: −0.0777-0.1543, including 0). In the specific pathways, IGR showed a significant positive regulation on miR-34a (*β* = 0.6982, *p* = 0.0401) but had no significant regulatory effect on miR-126-3p (β = −0.3018, *p* = 0.5033). Notably, after adjusting for miR-34a, the direct effect of IGR on cognitive impairment was no longer significant (β = 0.4025, *p* = 0.1063), indicating a complete mediation. High expression of miR-34a significantly increased the risk of cognitive impairment (β = 0.6473, *p* < 0.001), and high expression of miR-126-3p also showed a protective effect, significantly reducing the risk of cognitive impairment (β = −0.1261, *p* = 0.0038). However, unlike miR-34a, miR-126-3p was not regulated by IGR status and therefore did not serve as a mediator in this pathway Figure 2. Path diagram of mediating effect analysis.

### Comparative analysis of the diagnostic efficacy of miR-34a-5p and miR-126-3p

**Figure 3:**
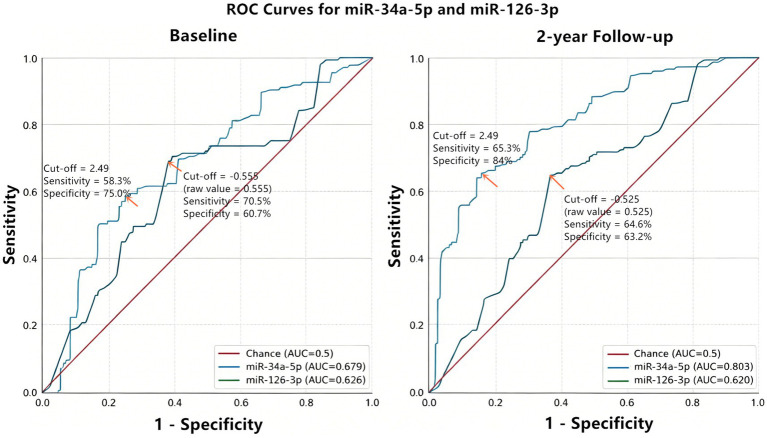
Diagnostic efficacy ROC curves of miR-34a-5p and miR-126-3p at baseline and the 2-year follow-up.

In this study, the diagnostic performance of miR-34a-5p and miR-126-3p was evaluated at baseline and at the 2-year follow-up using the Youden index to determine the optimal cut-off value (Figure 3).

The area under the curve (AUC) of miR-34a-5p was found to be 0.679 (95% CI: 0.617–0.740) at baseline, which was significantly improved to 0.803 (95% CI: 0.752–0.853) at 2-years. The Youden index revealed an optimal cut-off value of 2.490 at both time points. At baseline, this point was found to have a specificity of 75% and a sensitivity of 58.3%. At Follow-up the Diagnostic Performance Improved With the Same Cut-Off Yielding Sensitivity of 65.3% and Specificity of 84.0% (Youden Index, J = 0.493).

As values were reversed for analysis due to an overall negative correlation between miR-126-3p and cognitive impairment, the AUC was 0.626 at baseline and 0.620 2 years later. At baseline the optimal cut-off −0.555 corresponding to a sensitivity of 70.5% and specificity of 60.7%. At follow-up, −0.525 was the optimal cut-off with a sensitivity of 64.6% and a specificity of 63.2% (Youden index, J = 0.278).

### Functional enrichment and pathway analysis of miR-34a-5p target genes

**Figure 4:**
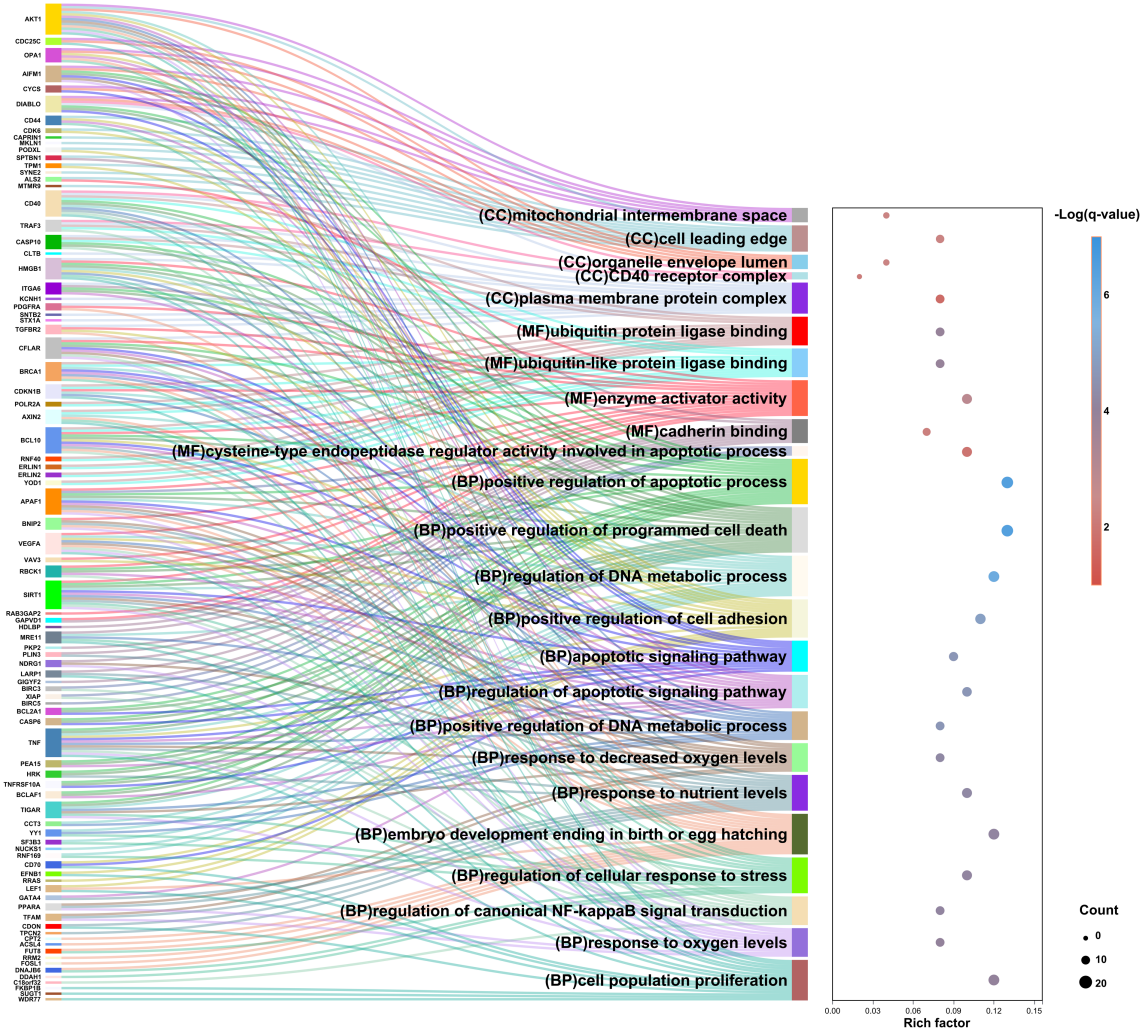
GO functional association network diagram (left) and GO functional enrichment bubble plot (right) for miR-34a-5p target genes.

GO functional enrichment analysis of miR-34a-5p target genes (Figure 4) revealed: In biological processes, core enriched terms included positive regulation of apoptotic process, positive regulation of programmed cell death, apoptotic signaling pathway, etc. (−Log (*q*-value) > 6), suggesting miR-34a-5p may participate in the pathology of cognitive impairment by regulating apoptosis-related biological processes. In molecular functions, ubiquitin protein ligase binding and ubiquitin-like protein ligase binding were key terms, reflecting the role of its target genes in protein degradation and homeostasis regulation. In cellular components, enrichment was found in mitochondrial intermembrane space, cell leading edge, etc., suggesting the subcellular localization of its target genes is related to mitochondrial function and dynamic changes in cell structure.

In the left panel, the left nodes represent target genes of miR-34a-5p and the right nodes represent significantly enriched GO terms (annotated by CC/BP/MF as classification labels); the connecting lines represent gene-function associations. In the right panel, the X-axis represents RichFactor (enrichment density), the Y-axis represents –Log (*q*-value) (significance), bubble size represents the number of target genes, and the color corresponds to the –Log (*q*-value).

**Figure 5:**
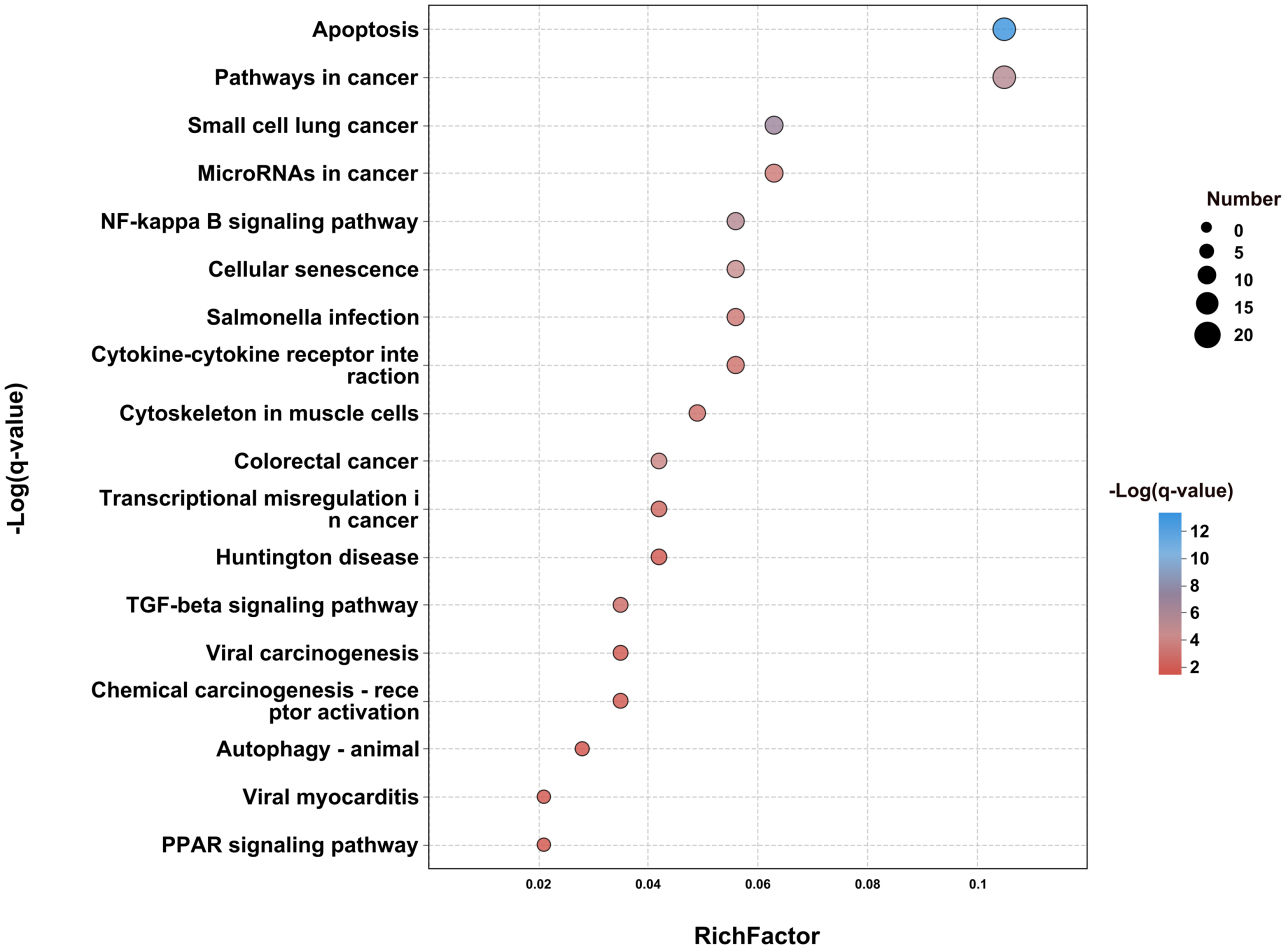
KEGG pathway enrichment bubble plot for miR-34a-5p target genes.

Further KEGG pathway enrichment analysis (Figure 5) showed that miR-34a-5p target genes were significantly enriched in the Apoptosis pathway (−Log (*q*-value) > 12, RichFactor ≈ 0.09), and also involved cancer-related pathways (e.g., Pathways in cancer), NF-κB signaling pathway, etc. Among these, the Apoptosis pathway showed the highest enrichment degree (both –Log (*q*-value) and RichFactor were the highest among all pathways), suggesting this pathway is one of the core molecular pathways through which miR-34a-5p regulates CSVD cognitive impairment.

The Y-axis represents –Log (*q*-value) (pathway significance), and the X-axis represents RichFactor (enrichment density); bubble size represents the number of target genes in the pathway, and the color corresponds to the –Log (*q*-value).

## Discussion

CSVD, as one of the main causes of cognitive impairment in the elderly, accounts for approximately 50% of global dementia cases (Ren et al., 2022). The association between its imaging features and cognitive decline has been widely confirmed (Markus et al., 2025). The prevalence of VaD among people aged 60 and above in China reaches 1.6%, making it the second most common type of dementia after Alzheimer’s disease (Jia et al., 2020a; Jia et al., 2020b). However, the current clinical management of CSVD cognitive impairment faces two core challenges: firstly, the lack of efficient molecular markers for early diagnosis—traditional diagnosis relies on MRI and neuropsychological scales, but MRI is costly and has low accessibility in primary healthcare institutions, and VCI is easily misdiagnosed based solely on scales and clinical symptoms; secondly, the intervention time window remains unclear. Previous research has mostly focused on the impact of T2DM on CSVD cognitive function. Approximately 40% of T2DM present with imaging markers of CSVD, which are positively correlated with the severity of cognitive decline (Unnikrishnan et al., 2025; Teng et al., 2021). However, insufficient attention has been paid to the pre-diabetic state of impaired glucose regulation (IGR), which may lead to delayed intervention.

Moghadasi et al. (2024) confirmed that miR-34a-5p can regulate peroxisome proliferator-activated receptor gamma (PPARγ) expression by inhibiting DNA methyltransferase (DNMT) activity, exacerbating insulin resistance and microvascular damage, which is highly consistent with the core pathology of CSVD (Markus et al., 2025). A retrospective study by Lakatos et al. (2025) on 94 patients with isolated acute microvascular lacunar infarction confirmed a clear association between chronic hyperglycemia and cerebral microangiopathy. Using transfer function analysis to assess dynamic cerebral autoregulation (dCA) parameters (phase, gain), they found that elevated HbA1c levels were significantly associated with a marked increase in gain at very low frequencies (0.02–0.07 Hz) (*p* = 0.02) and a trend towards increase at low frequencies (0.07–0.20 Hz) (*p* = 0.07), suggesting that long-term blood glucose dysregulation may promote the occurrence of microvascular cerebral infarction by increasing vascular stiffness and impairing dCA function. A prospective community-based study (CIRCS study) by Imano et al. (2018) showed that compared to normoglycemic individuals, pre-diabetic patients had a significantly increased risk of lacunar infarction, with a multivariable-adjusted hazard ratio (HR) of 2.02 (95% CI: 1.19–3.43), confirming that the IGR stage significantly increases the risk of lacunar infarction, a core pathological change of CSVD. The above studies suggest that the IGR stage may have already initiated miR-34a-5p-mediated abnormal epigenetic regulation of PPARγ, and such alteration, superimposed with HbA1c-related dCA impairment, collectively increases the risk of CSVD cognitive impairment. This also supports the rationality of our study selecting the IGR population as the intervention entry point and implies that the hyperglycemic state and insulin dysregulation during the IGR period may aggravate cognitive function damage in CSVD patients.

This study enrolled a cohort of CSVD patients, including those with and without impaired glucose regulation (IGR), and selected microRNAs related to cellular senescence (miR-34a-5p) and vascular endothelial function (miR-126-3p) (Yu et al., 2019) as candidate molecular markers for systematic analysis. The results of multivariate logistic regression analysis showed that miR-34a-5p expression was positively correlated with the risk of cognitive impairment (Exp(B) = 1.182, 95% CI: 1.058–1.319, *p* = 0.003), while miR-126-3p was negatively correlated (Exp(B) = 0.787, 95% CI: 0.683–0.908, *p* = 0.001), consistent with previous research findings (Moghadasi et al., 2024; Lakatos et al., 2025). Further analysis showed that miR-126-3p expression decreased in a gradient manner with worsening cognitive impairment (baseline: *H* = 21.793, *p* < 0.001; 2-year follow-up: *H* = 19.783, *p* < 0.002) and can serve as a reference indicator for the severity of cognitive impairment. miR-34a-5p was significantly correlated with the “normal/abnormal” status of cognitive function (baseline: *H* = 30.404, *p* < 0.001; 2-year follow-up: *H* = 31.465, *p* < 0.01) and is more suitable for the differential diagnosis of cognitive impairment.

Mediation effect analysis identified a potential correlative pathway. IGR was positively correlated with elevated miR-34a-5p expression (*β* = 0.698, *p* = 0.01), and increased miR-34a-5p expression was associated with a higher risk of cognitive impairment (*β* = 0.647, *p* < 0.01), while IGR exerted no significant regulatory effect on miR-126-3p (*β* = −0.302, *p* = 0.503). In light of Moghadasi et al.’s (2024) study, it is speculated that the combination of elevated fasting blood glucose (FBG) and long-term hyperglycemic state (which is reflected as elevated HbA1c) at the IGR stage lead to microvascular changes via impaired dCA function (Lakatos et al., 2025) and increase risk of lacunar infarct (Imano et al., 2018). Simultaneously, hyperglycemia can trigger the upregulation of miR-34a-5p, which enhances PPARγ promoter methylation via the inhibition of DNMT activity (Moghadasi et al., 2024) which leads to lowered PPARγ expression and aggravation of insulin resistance and vascular endothelial damage. It is shown that miR-34a-5p could be mediated molecules responsible for enhancing neural death and blood–brain barrier damage by inhibiting anti-apop-totic genes targeting CFLAR and down-regulating others like SIRT1 that provides antioxidant protection (Liu et al., 2024) leading to cognitive impairment. This conclusion is consistent with Quillian et al. (2025) showing that adolescents with IGR have abnormal glucose metabolism, which contributes to cognitive decline and is associated with central insulin sensitivity independently.

A key finding of this study is the temporal evolution in the diagnostic accuracy of miR-34a-5p, which distinguishes it from miR-126-3p. At baseline, both markers showed moderate diagnostic utility. However, miR-34a-5p demonstrated a substantial improvement at the 2-year follow-up, with its area under the curve (AUC) increasing from 0.679 to 0.803. This progression was accompanied by a balanced sensitivity of 65.3% and a specificity of 84.0% at the optimal cut-off of 2.49, underscoring its potential as a prognostic marker aligned with the chronic progression of CSVD. In contrast, miR-126-3p (analyzed with reversed values) showed relatively stable but limited performance, with an AUC of 0.626 at baseline and 0.620 at follow-up. Despite a sensitivity of 64.6% at follow-up, its specificity remained modest at 63.2%, constraining its standalone diagnostic value. Therefore, miR-34a-5p emerges as a biomarker that not only aids in identifying cognitive impairment but also serves as a more robust predictor over time, reflecting cumulative pathological burden, whereas miR-126-3p’s utility may be more confined to screening due to its specificity limitations. This conclusion is supported by the findings of Liu et al. (2024), who reported that combining SIRT1 mRNA and miR-34a-5p improved classification accuracy, achieving an AUC of 0.846. The observed dynamic improvement in miR-34a-5p’s accuracy is in accordance with the progressive nature of CSVD cognitive impairment, the cumulative effects of hyperglycemia-related microangiopathy, and the epigenetic regulatory role of miR-34a-5p. Jia et al. (2024) found that generalized fractional anisotropy (GFA) metrics, reflecting structural connectivity, continuously decreased with CSVD progression and correlated with cognitive scores. The expression changes of miR-34a-5p can synchronously reflect the cumulative process of “hyperglycemic microvascular damage – epigenetic regulatory abnormalities [e.g., PPARγ methylation (Moghadasi et al., 2024)] – neuronal apoptosis – cognitive decline.” The enhancement in its diagnostic efficacy may stem from these continuous molecular regulatory effects during disease progression, making it a biomarker valuable for both early diagnosis and long-term prognosis assessment. Its diagnostic performance is comparable to the artificial intelligence-assisted oculo-gait assessment model developed by Chen et al. (2024) (AUC 0.787–0.810) but is more convenient and cost-effective, rendering it more suitable for promotion and application in primary healthcare settings.

To preliminarily explore the potential molecular correlation by which miR-34a-5p mediates IGR-induced cognitive impairment in CSVD, this study obtained a high-confidence target gene set through miRWalk screening and miRTarBase validation. Metascape enrichment analysis revealed that miR-34a-5p target genes were not only significantly enriched in classical pathways such as apoptosis (CASP10, AIFM1), oxidative stress (SIRT1, TIGAR), and synaptic regulation (SNTB2, VEGFA) (with apoptosis as the core) but also included intersecting genes directly related to cerebral small vessel wall cell function: ACTA2 (a marker of arterial smooth muscle contraction) and KCNJ8 (key for pericyte vascular tone regulation). The functions of these two genes have been confirmed in CSVD pathogenesis research (Markus et al., 2025), and both have predicted potential binding sites for miR-34a-5p (ΔG < −19 kcal/mol, high binding confidence). This result is speculatively consistent with the pathology of CSVD cognitive impairment and IGR injury mechanisms. Markus et al. (2025) pointed out that chronic ischemia in CSVD leads to blood–brain barrier disruption and neuronal apoptosis, and it is speculated that miR-34a-5p may exacerbate ischemia by targeting and inhibiting ACTA2 (weakening small artery contractile function) and KCNJ8 (disrupting pericyte-endothelial communication). This process conforms to the pathological logic that abnormal cerebral small vessel wall cell function causes ischemia. Meanwhile, miR-34a-5p may activate the CASP10 apoptotic cascade, may inhibit SIRT1 antioxidant function, and may amplify the “hyperglycemia - microvascular damage - neuronal loss” effect, which aligns with the conclusions of Moghadasi et al. (2024) (miR-34a regulating PPARγ causing metabolic disorders), (Lakatos et al., 2025) (HbA1c impairing cerebral blood flow regulation), and (Imano et al., 2018) (IGR increasing lacunar infarction risk). Moreover, the enrichment of synaptic regulatory genes such as SNTB2, combined with the findings by Xu et al. (2018) that miR-34a regulates NMDA receptors affecting synaptic strength, suggests a possible core regulatory chain: “IGR → increased miR-34a-5p → inhibition of ACTA2/KCNJ8 + activation of apoptosis/oxidative stress → abnormal cerebral small vessel function + synaptic dysfunction → CSVD cognitive impairment.” Significantly, all inferences of the molecular mechanism in the present study were made from data present in the literature. The precise molecular mechanisms will need further verification through follow-up *in vitro* cell experiments and *in vivo* animal studies, which are currently ongoing in our team.

This study is not without limits. Firstly, selection bias can be introduced by the single-center sample which can reduce the generalizability of the results; future multi-center cohort studies are needed to confirm the universal applicability of the conclusions. Second, the present study has not yet combined the imaging markers of CSVD (white matter hyperintensity volume, Fazekas grade, etc.) with the expression analysis of miR-34a-5p target genes. A previous study showed that structural changes of the amygdala were related to CSVD-related cognitive impairment. Higher diagnostic efficiency may be achieved by combining various markers (Cheng et al., 2024). The GO/KEGG pathway enrichment analysis in that study only represents a database-based prediction and has not been experimentally validated. To validate the regulatory pathways potentially involving miR-34a-5p and its target genes, we are presently conducting relevant basic experiments. There is a lack of interventional data. It remains to be validated through future clinical trials whether downregulating miR-34a-5p expression or optimizing blood glucose levels can enhance cognitive function by reversing the above regulatory pathways.

Notwithstanding these limitations, this study yields preliminary evidence on core conclusions: ① IGR may represent a novel intervention time window for CSVD cognitive impairment, as the pathological abnormalities of cerebral small vessels have developed by this stage; ② miR-34a-5p may possess high potential as a superior biomarker (AUC = 0.803 at 2-year follow-up) with diagnostic and prognostic values, while miR-126-3p may help to assess cognitive impairment severity; ③ miR-34a-5p may potentially mediate the regulation of IGR on CSVD cognitive function by targeting genes related to apoptosis, oxidative stress, and synaptic function.

To summarize, this study first provides clinical evidence linking IGR status, dysregulation of miR-34a-5p and cognitive impairment in CSVD patients. These conclusions may point to the IGR stage as the time window for vigilance. MiR-34a-5p could be a good candidate biomarker for risk stratification. Future studies can explore their usefulness for precise screening and early intervention.

## Data Availability

The original contributions presented in the study are included in the article/supplementary material, further inquiries can be directed to the corresponding author.
